# How has the flu virus infected the Web? 2010 influenza and vaccine information available on the Internet

**DOI:** 10.1186/1471-2458-13-83

**Published:** 2013-01-29

**Authors:** Loredana Covolo, Silvia Mascaretti, Anna Caruana, Grazia Orizio, Luigi Caimi, Umberto Gelatti

**Affiliations:** 1 Department of Medical and Surgical Specialties, Radiological Sciences and Public Health, University of Brescia, Viale Europa 11, 25123, Brescia, Italy; 2 Post-graduate School of Public Health - University of Brescia, Viale Europa 11, 25123, Brescia, Italy; 3 Department of Medical Prevention - Brescia Local Health Authority, Viale Duca degli Abruzzi 15, 25124, Brescia, Italy; 4 “Quality and Technology Assessment, Governance and Communication Strategies in Health Systems”, Study and Research Centre - University of Brescia, Viale Europa 11, 25123, Brescia, Italy

**Keywords:** Internet, Influenza vaccine, Public health, Information

## Abstract

**Background:**

The 2009–10 influenza pandemic was a major public health concern. Vaccination was recommended by the health authorities, but compliance was not optimal and perception of the presumed associated risks was high among the public. The Internet is increasingly being used as a source of health information and advice. The aim of the study was to investigate the characteristics of websites providing information about flu vaccine and the quality of the information provided.

**Methods:**

Website selection was performed in autumn 2010 by entering eight keywords in two of the most commonly used search engines (Google.com and Yahoo.com). The first three result pages were analysed for each search, giving a total of 480 occurrences. Page rank was evaluated to assess visibility. Websites based on Web 2.0 philosophy, websites merely displaying popular news/articles and single files were excluded from the subsequent analysis. We analysed the selected websites (using WHO criteria) as well as the information provided, using a codebook for pro/neutral websites and a qualitative approach for the adverse ones.

**Results:**

Of the 89 websites selected, 54 dealt with seasonal vaccination, three with anti-H1N1 vaccination and 32 with both. Rank analysis showed that only classic websites (ones not falling in any other category) and one social network were provided on the first pages by Yahoo; 21 classic websites, six displaying popular news/articles and one blog by Google. Analysis of the selected websites revealed that the majority of them (88.8%) had a positive/neutral attitude to flu vaccination. Pro/neutral websites distinguished themselves from the adverse ones by some revealing features like greater transparency, credibility and privacy protection.

**Conclusions:**

We found that the majority of the websites providing information on flu vaccination were pro/neutral and gave sufficient information. We suggest that antivaccinationist information may have been spread by a different route, such as via Web 2.0 tools, which may be more prone to the dissemination of “viral” information. The page ranking analysis revealed the crucial role of search engines regarding access to information on the Internet.

## Background

During the 2009–10 flu pandemic, compliance with preventive measures proposed by health authorities was not optimal. The presumed risks associated with vaccination attracted mass media attention, and risk perception among the public was high. Concerns regarding the population-based public health response to this pandemic focused on the need for more effective communication; a BMJ editorial cited an urgent need to restore public trust before the next pandemic comes along [1].

The Internet is increasingly used as a “trusted” source of health-related information, although our knowledge regarding the role of this source in shaping behaviour during a pandemic event is largely unknown. Of particular interest is the role of search engine algorithms, which mainly determine content visibility and user accessibility. Previous studies have investigated the role of different search terms in determining the search engine results. For example, keyword “vaccination” was found to lead to a greater amount of content with an adverse attitude towards vaccination compared to “immunization”, since the antivaccinationists have a strong philosophical preference for the former [2,3].

Recent contributions have pointed out that research on online vaccine information is quite rare and not up to date [4], although there is evidence that exposure to critical websites is associated with changes in risk perception [5]. Previous studies have explored the contents of antivaccinationist websites, analysing their statements and arguments [6-8], and the prevalence of quality markers in websites dealing with vaccination [9]. Other studies have analysed *YouTube* contents regarding immunization [10], including H1N1 influenza [11] and focused on the Internet’s influence on risk perception from a psychological perspective [12]. Regarding the specific issue of information about flu vaccination on the Internet, one study performed a content analysis of the information provided by the social network *Twitter* during the H1N1 outbreak [13]; another dealt with the probability of finding WHO recommendations on swine flu prevention [14]. A recent study has even investigated psychological and demographic factors associated with H1N1 vaccination, highlighting the critical role of communication strategies in shaping behaviour [15].

On the basis of the above considerations, this study aimed to evaluate online content regarding vaccination right after the 2009–10 pandemic in order to understand the role that Internet may have had in shaping people’s risk perception. To achieve this objective, we conducted an overall assessment of the websites providing flu vaccine information by evaluating their visibility (by rank analysis), and integrating this with a content analysis. Possible differences in the results provided by different search engines were also assessed.

## Methods

### Website selection

Website selection was performed, from an Italian IP address, in autumn 2010, via the two most commonly used search engines: Google.com and Yahoo.com [16]. Eight key phrases (“flu vaccine”, “flu vaccination”, “flu immunization”, “flu shot”, “influenza vaccine”, “influenza vaccination”, “influenza immunization” and “influenza shot”) were entered and the first three pages of results (each showing ten occurrences) were downloaded for each of them, giving a total of 480 occurrences. The selected key phrases were chosen by the authors assuming that an average Internet user would likely use them to make simple searches on the Web regarding influenza immunization.

The preliminary exclusion criteria were:

websites not available in English;

websites requiring registration for access;

Web pages merely containing links to other pages (portals);

websites which did not lead us to the research content within three clicks of the occurrence;

websites dealing with non-human-related matters (e.g. veterinary issues);

websites targeting health professionals only.

The advanced exclusion criteria were:

websites based on web 2.0 philosophy (e.g. blogs, social networks, communities, forums);

websites displaying popular news/articles;

single files (e.g. pdf, ppt).

We considered as “classic websites” those not falling in any of the above categories.

The search engine results were immediately saved in order to avoid variation in ranking. Web pages occurring more than once were only taken into account the first time.

The websites selected when applying both preliminary and advanced exclusion criteria were analysed. To allow a comprehensive rank assessment, only preliminary exclusion criteria were applied to the search engine results.

### Rank analysis

Page rank was assessed for all the occurrences on the first page only (corresponding to the first ten results) for Google and Yahoo which fitted the preliminary exclusion criteria. The results provided by the two search engines were then compared.

### Website coding

The websites selected when applying both preliminary and advanced exclusion criteria underwent a two-step analysis, of the website itself and of the information provided.

All the websites were analysed by one author. Critical points were discussed with the other authors and differences were resolved by consensus.

#### 1. Analysis of the website

1a. *Classification of adverse vs. pro/neutral websites*

Websites were considered adverse if they explicitly recommended that users should not comply with public health recommendations, suggested users try alternative preventive methods, declared that evidence about vaccine effectiveness/safety is inadequate, or overemphasized the risks associated with vaccination. Web pages not fitting such criteria were considered “pro/neutral” [3,17].

1b. *Comparison of adverse vs. pro/neutral websites with regard to the WHO’s “Good Information Practice Essential Criteria for Vaccine Safety Web Sites”*

Website analysis was performed on the basis of the WHO’s “Good Information Practice Essential Criteria for Vaccine Safety Web Sites” [18], partially simplified in respect of the original version but keeping the essential criteria. It specifically regarded the following:

general information: extension and type of website;

credibility: mission of site, disclosure of ownership or source, transparency of sponsorship, accountability to users, data protection and responsible partnering;

accessibility;

design: logical organisation, ease of navigation, consistent plan and professional presentation;

content: authority of sources, accuracy, currency and review process.

For each of the above we assessed whether the selected websites satisfied the specific requirements proposed by the WHO (cited in full in Table 1) or they did not and compared websites with opposing attitudes towards flu vaccination.

**Table 1:** Website analysis according to the WHO’s “Good Information Practice Essential Criteria for Vaccine Safety Web Sites”

**Website analysis criteria**	**Pro/neutralWebsites % (N=79)**	**Adverse Websites % (N=10)**	**p**
**GENERAL INFORMATION**			
**Website type**			
-Association/scientific society	8.9	10	-
-Pharmaceutical company	5.1	0	-
-Hospital/clinic/health facility	16.5	0	-
-Dictionary/encyclopaedia	2.5	0	-
-Online pharmacy	1.3	0	-
-Government website	32.9	0	-
-Academic website	6.3	0	-
-Other	26.6	90	-
**CREDIBILITY**			
**Mission of website**			
Website mission declared	96.2	70	0.0018
Public to whom information is addressed declared	96.2	70	0.0018
Content adheres to website mission (if mission is expressed, P= 76; A=7)*	100	85.7	0.0009
**Disclosure of ownership/source**			
Owner’s name (person or organisation) declared	96.2	80	0.036
Owner’s address declared	83.5	60	ns
Owner’s credentials/status declared	93.7	70	0.0137
If owner is an organisation, what type of organisation (if credentials are declared, P=74; A=7)*			
• Business organisation	35.1	0	-
• Governmental organisation	33.8	0	-
• Non-profit organisation	13.5	42.9	-
• Other	17.6	57.1	-
Presence of affiliations, partnerships, alliances	31.7	10	ns
Type of affiliation/ partner/ alliance (if present, P=25; A=1 )*			
• Government organisation	40	0	-
• Public health agency	32	0	-
• Associations/groups	20	0	-
• Clinic/hospital	24	0	-
• Medical/health information websites	36	0	-
• Other	56	100	-
List of members of Editorial Board, Advisory Board, Board of Directors (at least one) and their credentials	63.3	50	ns
**Transparency of sponsorship**			
Sources of funding declared	70.9	40	0.0496
Presence of advertising	30.4	20	ns
Declaration/description of advertising policy (if advertising present, P=24; A=2 )*	83.3	50	ns
Contents aimed at promotion and online selling (products/services)	41.8	60	ns
Contents aimed at promotion/online selling clearly visible (if present, P= 33; A=6)*	97	83.3	ns
Absence of conflict of interests is declared	16.5	0	ns
Who declares absence of conflict of interests (if declared, P=13 )*			
- Sponsor/Donor	61.5	0	-
- Partner/Affiliated	7.7	0	-
- Author/Responsible	69.2	0	-
**Accountability to users**			
Owner can be contacted by telephone/e-mail/electronic form/fax (at least one)	97.5	90	ns
Contacts are accessible on the homepage and also on the other Web pages (if owner can be contacted, P=77; A=9)*	96.1	55.6	0.0000
Interactive communication is possible (e.g. chat room, forum, community, etc.)	36.7	30	ns
Information about moderator on the page dedicated to interaction (if interactive communication is possible, P=29; A=3)*	17.2	66.7	0.0487
Possible inaccuracy of information published in the posts is declared (if interactive communication is possible, P=29; A=3)*	27.6	33.3	ns
**Data protection**			
User is informed about possible use of his personal data	88.6	20	0.0000
Presence of privacy statement or confidentiality policy page	91.1	40	0.0000
Data firewall from non-authorized accesses	68.4	20	0.0029
**Responsible partnering**			
Notification when leaving the website	7.6	0	ns
**ACCESSIBILITY**			
Ease of access to the website	100	100	ns
it’s possible to download programs to open files (audio, video, pdf)	40.5	10	0.0599
Presence of internal search engine	86.1	70	ns
Return to previous websites is possible	98.7	100	ns
Absence of “broken links”	86.1	90	ns
Presence of “reserved areas”	41.8	20	ns
Presence of copyrighted information	87.3	50	0.003
Information about legal use/distribution of copyrighted material (if present, P= 69; A=5)*	76.8	80	ns
Website availability in different languages	44.3	10	0.0373
It’s possible to change fount	31.7	10	ns
**DESIGN**			
Logical/ordered access to information	100	90	0.0047
Ease of Web surfing: at least two of site map/index/help function/FAQ page/internal search engine	79.8	40	0.006
Consistent design (layout, font, logos, icons, etc.	93.7	60	0.0009
Professional appearance/pleasing aesthetics	94.9	50	0.0000
**CONTENT**			
**Authority of sources**			
Author is declared for each content	27.9	70	0.0074
People in charge team for website in general is declared	50.6	10	0.0152
Authors’ credentials are declared	57	80	ns
What kind of credentials (if they’re declared, P= 45; A=8)*			
• Medical	95.6	62.5	0.0032
• Other	28.9	50	ns
Presence of quality marks/awards/certifications	41.8	10	0.0514
**Accuracy**			
It’s possible to contact author/person in charge for website content	12.7	40	0.0253
**Currency**			
Date of Web page content compilation is declared	22.8	10	ns
Date of last Web page updating is declared	55.7	0	0.0009
Date of last website updating is declared	8.9	0	ns

Notes:

Percentages were calculated using as denominators the total number of pro/neutral websites (N=79) and the total number of adverse websites (N=10), except when marked *.

* : the denominator is the number of websites with the feature in question.

P: websites with pro/neutral attitude to flu vaccine.

A: websites with adverse attitude to flu vaccine.

ns: not statistically significant.

#### 2. Analysis of the information provided

2a. *Analysis of the information provided by pro/neutral websites*

The information provided by pro/neutral websites was analysed according to the content analysis method [19], using a codebook that enabled the following information to be collected: type of flu vaccine (seasonal flu vaccine, H1N1 flu vaccine, or both: if not specified, seasonal flu vaccine was considered), general information, administration route, doses, administration period, indications, effectiveness, contraindications, benefits and risks.

For each of the above we evaluated the presence of information, completeness and adherence to the gold standard - represented by the “WHO Position Paper on Influenza” [20] and “Vaccine for pandemic H1N1 2009” [21] - and whether scientific bibliographical references were provided.

Websites providing information on both types of vaccine were analysed twice: with regard to information about the H1N1 vaccine and to information about the seasonal vaccine.

2b. *Analysis of the information provided by adverse websites*

A qualitative approach was used for analysing the information provided by adverse websites, to assess the presence of arguments used by the authors to support their attitude.

#### Statistical analysis

Data on the comparison of adverse vs. pro/neutral websites with regard to the WHO criteria and on the comparison of information about seasonal and H1N1 flu vaccine were analysed using STATA (Stata Statistical Software Release 8.0, 2003; Stata Corporation, College Station, TX). We adopted a descriptive approach and calculated statistical significance using the X2 test, rejecting the null hypothesis below a p-value of 0.05.

## Results

**Figure 1:**
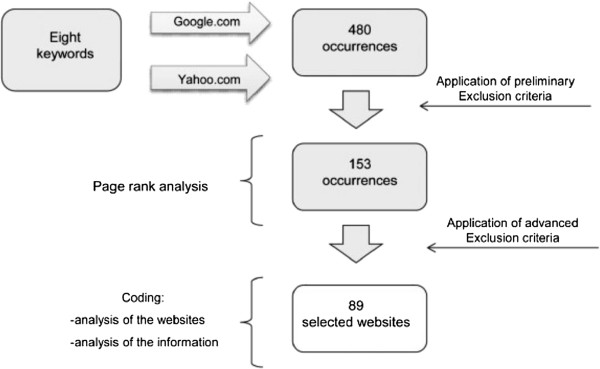
Flow-chart of the analyses performed.

An overall flow-chart of the analysis process is shown in Figure 1.

### Website selection

Using the above preliminary and advanced exclusion criteria, we selected 89 websites providing information about flu vaccination.

### Rank analysis

A total of 49 different occurrences fitting the preliminary inclusion criteria were shown on the first search engine page for each key phrase.

Differences were revealed with regard to the kind of website shown by the two search engines: whereas Yahoo only showed classic websites and one based on Web 2.0 philosophy, Google ranked on the first result page 21 classic websites, six displaying popular news or articles and one based on Web 2.0 philosophy. In addition, whereas Google always ranked Wikipedia first, Yahoo ranked four websites: three with a .gov extension (cdc.gov, csm.gov, flu.gov) and Wikipedia. Two adverse websites appeared on the first page of Google and only when entering two key phrases (“flu shot” and “flu vaccine”), while no adverse website appeared on the first page of Yahoo.

**Figure 2:**
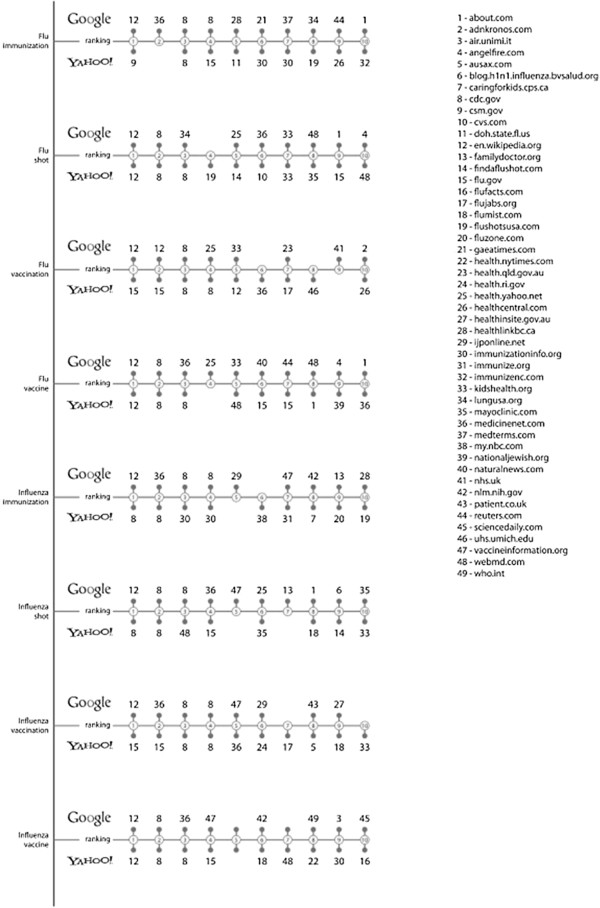
**Page Rank Analysis.** Figure 2 shows the positions of occurrences obtained on the first pages of both search engines when entering the eight key phrases. Blank spaces correspond to non-eligible websites.

Figure 2 shows the positions of occurrences on the first page of both the search engines when the eight key phrases were entered. Blank spaces correspond to non-eligible websites.

### Website coding

#### 1. Analysis of the website

1a. *Classification of adverse vs. pro/neutral websites*

Analysis of the websites revealed that the majority (88.76%; n 79) had a positive/neutral attitude to flu vaccination, and only ten of them (11.23%) were adverse.

1b. *Comparison of adverse vs. pro/neutral websites with regard to the WHO’s “Good Information Practice Essential Criteria for Vaccine Safety Web Sites”*

The most frequent extension was *.com* (60% of adverse and 35.4% of pro/neutral websites, p>0.05), followed by .*org* (10% of adverse and 16.5% of pro/neutral websites, p>0.05). The extensions .*gov* and .*net* characterized pro/neutral websites only (16.5% and 2.5% respectively, p>0.05; data not shown).

Remarkable differences were noted between websites with opposing attitudes. The results of this comparison are shown in Table 1.

#### 2. Analysis of the information provided

2a. *Analysis of the information provided by pro/neutral websites*

Of the pro/neutral websites, 51 dealt with seasonal flu vaccine only, three with anti-H1N1 flu vaccine only, and 25 both of them.

Table 2 shows the percentages of websites dealing with seasonal and H1N1 flu vaccine which offer general information about the vaccine and notions about administration routes, doses, vaccination period, indications, effectiveness, contraindications, benefits and risks. The percentages of websites providing comprehensive and coherent information in relation to gold standard and scientific bibliographic references are reported for each of these topics.

2b. *Analysis of the information provided by adverse websites*

The analysis was performed on nine of the ten selected classic websites with an adverse attitude to flu vaccination, since one of them disappeared from the Web after selection.

**Table 2:** Analysis of the information provided by the websites. Comparison of information about seasonal and H1N1 flu vaccine

**Website analysis criteria**	**Seasonal vaccine % (N=76)**	**H1N1 vaccine % (N=28)**	**p**
**General information about vaccine**	86.8	75	ns
Gold standard adherence	100	100	ns
Completeness in relation to gold standard	21.2	57.1	0.0017
Scientific bibliographical references	56.1	71.4	ns
**Information about administration routes**	64.5	57.1	ns
Gold standard adherence	100	100	ns
Completeness in relation to gold standard	61.2	68.8	ns
Scientific bibliographical references	59.2	68.8	ns
**Information about doses**	59.2	64.3	ns
Gold standard adherence	100	100	ns
Completeness in relation to gold standard	42.2	83.3	0.0031
Scientific bibliographical references	62.2	83.3	ns
**Information about vaccination period**	84.2	57.1	0.0037
Gold standard adherence	100	100	ns
Completeness in relation to gold standard	32.8	56.3	ns
Scientific bibliographical references	53.1	68.8	ns
**Information about indications**	97.4	100	ns
Gold standard adherence	100	100	ns
Completeness in relation to gold standard	82.4	75	ns
Scientific bibliographical references	66.2	78.6	ns
**Information about effectiveness**	75	60.7	ns
Gold standard adherence	98.2	100	ns
Completeness in relation to gold standard	19.3	23.5	ns
Scientific bibliographical references	54.4	76.5	ns
**Information about contraindications**	88.2	57.1	0.0005
Gold standard adherence	100	93.7	ns
Completeness in relation to gold standard	61.2	31.3	0.0304
Scientific bibliographical references	53.7	75	ns
**Information about benefits**	80.3	64.3	ns
Gold standard adherence	100	100	ns
Completeness in relation to gold standard	42.6	38.9	ns
Scientific bibliographical references	52.4	77.8	ns
**Information about risks**	85.5	64.3	0.0167
Gold standard adherence	100	100	ns
Completeness in relation to gold standard	38.5	55.6	ns
Scientific bibliographical references	56.9	66.7	ns

Notes:

Percentages were calculated using as denominators the number of websites dealing with the seasonal vaccine only + websites dealing with both vaccines, and the number of websites dealing with the H1N1 vaccine only + websites dealing with both vaccines.

ns: not statistically significant.

The websites dealt with several arguments, as shown in Table 3. Danger to human health related to vaccination was the most widely recurring one: 100% of the websites analysed provided descriptions of possible adverse reactions, often (55.5%) recounting dramatic personal experiences *(“After getting it, I became ill, not really bad but sort of nagging lingering feeling of not being well […] I woke up next morning with my left arm paralyzed…”).*

**Table 3:** Recurring arguments on websites with an adverse attitude to seasonal/H1N1 flu vaccine

**Website**	**Vaccine is dangerous**		**Vaccine is useless**	**Economic Interests; political control**	**Public ignorance**	**Inefficiency of traditional medicine; promoting alternative medicine**	**Flu is not a serious problem**	**Enlight- ened physician**	**Ethical reasons**	**Bizarre theories**
	**Adverse effects**	**Toxic consti- tuents**								
Jeffreywarber.com	X	X	X	X	X	X	X	X	X	
Drtenpenny.org	X	X	X	X	X	X		X		
Thinktwice.com	X		X		X					
Whale.org	X		X	X	X	X	X			X
Vaccinetruh.org	X	X	X	X						
Angelfire.com	X	X	X	X	X	X	X	X		
Naturalnews.com	X		X	X	X	X	X	X	X	X
Novaccine.com	X	X	X							
I-sis.org	X	X		X						

Six of them (66.7%) provided a list of the vaccine’s constituents and their respective risks for human health (*“Do you want any of these vaccine constituents in YOUR blood stream?*”).

Nearly all the websites (88.9%) argued that flu vaccination was useless, mainly because of poor matches between the viruses in the vaccines and those actually infecting people (*“…vaccines are being used as an ideological weapon. What you see every year as the flu is caused by 200 or 300 different agents with a vaccine against two of them. That is simply nonsense”).*

Seven of them (77.8%) stated that vaccination policy is driven by the enormous economic interests of pharmaceutical manufacturers, whose profits are boosted by exaggerated government claims.

According to six websites (66.7%), control over mass media leads to a substantial ignorance on the part of the public and influences personal choices, whereas everyone should be able to make informed decisions with regard to his or her own health.

Five websites (55.5%) dealt with doctors’ unreliability and the inefficiency of traditional medicine and/or invited readers to consider alternative medicine and homeopathic or natural remedies.

According to four websites (44.4%), the mass media and government are responsible for unjustified panic about flu, the severity of which is greatly overestimated (“*Aspirin kills 400% more people than H1N1 swine flu*”) and the same number of websites referred to “enlightened” physicians denouncing the system, some of them (22.2%) being authors themselves.

Two websites put forward ethical arguments, based on the use of pork products in vaccine manufacture *(“…to separate cells we have to use a pork product - pork trypsin - and a lot of people who would not ingest pork products for religious reasons are seduced without knowing it into violating their convictions…”*) and referred to alleged tests on homeless people in Poland.

Unusual theories were discussed by two authors: one claimed that seasonal flu vaccination greatly increases the risk of contracting H1N1 flu by weakening the immune system; the other described the 1918 “Spanish Influenza” as the “Frankenstein monster” created by American doctors with their vaccines.

In addition to recurring arguments, adverse websites showed other common features, such as the above-mentioned predilection for narrating personal experiences and a significant propagandistic attitude. On six websites (66.6%) we found an invitation to promote the anti-vaccination movement by involving new people, joining activist associations, writing articles for local newspapers, and selling alternative products and information packs online. One website even contained a link to an anti-vaccination song entitled “Don’t inject me”.

Only one article invited readers to discuss with their doctors whether they should get vaccinated.

## Discussion

According to the above findings, the majority of the selected websites had a positive/neutral attitude to flu vaccination. They distinguished themselves from the adverse ones by some revealing features: firstly, greater transparency, in relation to more frequent declaration of the sources of funding; secondly, greater credibility, since in a statistically higher percentage of cases explicitly declared the website mission and the public to whom the information is addressed. In addition, their content always adhered to their stated mission (not the same for adverse websites) and they more frequently declared the owner’s name and credentials. Furthermore, they were characterized by higher data and privacy protection and by more professional appearance and consistent design. Lastly, they showed a significantly higher amount of copyrighted information, which can be considered as an expression of responsibility for content.

The quality of the information provided by pro/neutral websites was generally satisfactory. In particular, adherence to the gold standard was optimal, whereas data about completeness were less satisfactory, especially on websites dealing with seasonal flu vaccine.

The presence of information about vaccination period, risks and contraindications was statistically higher for the seasonal flu vaccine compared to the H1N1 vaccine, so it is possible to assume that the set of information available on the Web about the former is more exhaustive and scientifically structured. Completeness of the information about contraindications was also better for the seasonal vaccine, while information about doses was more complete for the H1N1 vaccine.

Previous studies have pointed out that the influence of Internet information on risk perception, decision process and hence vaccination behaviour does not always act in a conscious way; the fact that individuals do not admit to considering the Internet a reliable source does not necessarily mean that it does not influence their choices [22,23]. Based on the above results, the way adverse websites were formulated seems to be consistent with the purpose of influencing the readers’ attitude towards vaccination. Some arguments were often repeated: danger of vaccine due to adverse affects and toxic constituents, economic interests driving vaccination policy (the “plot theory”), unreliability of institutional medicine. Furthermore, a narrative form widely prevailed. Such personal and emotional narration, frequently regarding children and performed between parents, has been proved to be a critical factor of the effects of antivaccinationist information [5,23], since it works on readers’ emotions and fears.

Search engine ranking refers to the position at which a particular occurrence appears in the results of a search engine query. As a consequence, it is closely connected with visibility to users.

Differences were highlighted in the kind of results provided by the search engines. In particular adverse websites were shown on the first page by Google only, and Yahoo results were characterized by a higher presence of institution (.gov extension) in the first positions, which we expect to contain a more reliable information [24].

Page rank is a crucial factor based on what can be called “the internet paradox”: although the Web contains virtually unlimited information, it has been observed that users generally do not go beyond the first page of search engine results and have a low tolerance of going in depth through what is retrieved [25].

One particular strength of this study is that we performed a broad-spectrum analysis on different levels: visibility according to page ranking, analysis of the websites and analysis of the information provided, choosing for websites with opposing attitudes to flu vaccination the methods which most suited their features.

One limitation of the study is intrinsic to the Internet: information on the Web is constantly changing, whereas we analysed the Web pages which were available at a particular time. Since we carried out website selection in autumn 2010, some months after the flu pandemic peaked, we realise that page rank may well have changed in the meantime, as the topic was becoming of less pressing actuality.

Secondly, our assessment of the information provided using different methods of analysis, based on the websites’ attitude towards flu vaccination, was due to the very nature of the information. Actually, there was an extreme difference in the way pro/neutral and adverse websites were structured. Adverse websites, far from providing a structured set of information, as the pro/neutral ones did, showed a prevalence of narrative form, rich in dramatic personal experiences which were generally related to adverse reactions to the vaccine. The use of a fixed analysis scheme would have prevented us from assessing the highly intriguing topics and theories used by antivaccinationist authors to support their attitude and spread it among the people.

Moreover, Web 2.0 was excluded from the analysis. This was due to its extremely dynamic structure, which requires specific analysis strategies and makes it impossible to univocally assign an attitude to several comments, made by as many authors, on the same Web page.

Web 2.0 is continuously expanding and it is becoming a common way to obtain health information. Even important health institutions such as the CDC use the new communication tools, like mobile phone applications, in order to spread information among the population. We therefore suggest that a possible future development of the research could interestingly extend it to this area of the Internet, also in view of strategically exploiting its potential by the public health authorities.

Furthermore, we decided to limit the research to the two most commonly used search engines (Google.com and Yahoo.com), although others, such as Bing.com and Ask.com, are also very popular [16], and to a limited set of key phrases.

## Conclusions

The vast majority of the websites analysed had a positive/neutral attitude towards flu vaccination and overall they provided satisfactory information. They also were characterized by a certain presence of reliable organisations and institutions, for example the CDC (Centers for Disease Control and Prevention) official website and Flu.gov (a government website managed by the U.S. Department of Health & Human Services). This naturally raises the question as to whether antivaccinationist information on the Web could take different routes, such as Web 2.0. If confirmed, this would entail a new challenge for the public health authorities. Promotion of prevention on the Internet by providing traditionally structured information may no longer be sufficient and the use of new strategies, such as Web 2.0 tools, may be more effective as they may be more likely to spread information via “viral” dissemination.

## Competing interests

The authors declare that they have no competing interests.

## Authors’ contribution

UG, LC, LC and GO conceived and designed the study. AC and SM acquired the data. UG, LC, AC and SM analysed and interpreted the data. All the authors were involved in drafting the manuscript and revising it critically and have given their final approval of the version for publication.

## Pre-publication history

The pre-publication history for this paper can be accessed here:

http://www.biomedcentral.com/1471-2458/13/83/prepub
